# Exoantigens of *Paracoccidioides* spp. Promote Proliferation and Modulation of Human and Mouse Pulmonary Fibroblasts

**DOI:** 10.3389/fcimb.2020.590025

**Published:** 2020-10-30

**Authors:** Débora de Fátima Almeida Donanzam, Tatiani Ayako Goto Donato, Karoline Haghata dos Reis, Adriely Primo da Silva, Angela Carolina Finato, Amanda Ribeiro dos Santos, Ricardo Souza Cavalcante, Rinaldo Poncio Mendes, James Venturini

**Affiliations:** ^1^ Faculdade de Medicina, Universidade Federal do Mato Grosso do Sul, Campo Grande, Brazil; ^2^ Faculdade de Medicina, Departamento de Doenças Tropicais e Diagnóstico por Imagem, UNESP, Botucatu, Brazil; ^3^ Faculdade de Ciências, UNESP, Bauru, Brazil

**Keywords:** pulmonary fibroblast, pulmonary fibrosis, paracoccidioidomycosis, cell response, growth factors

## Abstract

Paracoccidioidomycosis (PCM) is a systemic granulomatous fungal infection caused by thermally dimorphic fungi of the genus *Paracoccidioides*. Endemic in Latin America, PCM presents with high incidence in Brazil, Colombia, and Venezuela, especially among rural workers. The main clinical types are acute/subacute (AF) form and chronic form (CF). Even after effective antifungal treatment, patients with CF usually present sequelae, such as pulmonary fibrosis. In general, pulmonary fibrosis is associated with dysregulation wound healing and abnormal fibroblast activation. Although fibrogenesis is recognized as an early process in PCM, its mechanisms remain unknown. In the current study, we addressed the role of *Paracoccidioides* spp. exoantigens in pulmonary fibroblast proliferation and responsiveness. Human pulmonary fibroblasts (MRC-5) and pulmonary fibroblasts isolated from BALB/c mice were cultivated with 2.5, 5, 10, 100, and 250 µg/ml of exoantigens produced from *P. brasiliensis* (Pb18 and Pb326) and *P. lutzii* (Pb01, Pb8334, and Pb66) isolates. Purified gp43, the immunodominant protein of *P. brasiliensis* exoantigens, was also evaluated at concentrations of 5 and 10 µg/ml. After 24 h, proliferation and production of cytokines and growth factors by pulmonary fibroblasts were evaluated. Each exoantigen concentration promoted a different level of interference of the pulmonary fibroblasts. In general, exoantigens induced significant proliferation of both murine and human pulmonary fibroblasts (p < 0.05). All concentrations of exoantigens promoted decreased levels of IL-6 (p < 0.05) and VEGF (p < 0.05) in murine fibroblasts. Interestingly, decreased levels of bFGF (p < 0.05) and increased levels of TGF-β1 (p < 0.05) and pro-collagen I (p < 0.05) were observed in human fibroblasts. The gp43 protein induced increased TGF-β1 production by human cells (p = 0.02). In conclusion, our findings showed for the first time that components of *P. brasiliensis* and *P. lutzii* interfered in fibrogenesis by directly acting on the biology of pulmonary fibroblasts.

## Introduction

Paracoccidioidomycosis (PCM) is a systemic granulomatous fungal infection caused by thermally dimorphic fungi of the genus Paracoccidioides (Teixeira et al., 2009). The infection occurs after inhalation of conidia or mycelia fragments that reach the lungs and morphologically switch to yeast forms (Restrepo et al., 2012). Clinically, PCM is mainly of two types, acute/subacute form (AF) and chronic form (CF) (Mendes et al., 2017). CF is the most common with clinical manifestations predominantly in the lungs and upper aerodigestive tract (Shikanai-Yasuda et al., 2017). Most CF-PCM patients exhibit pulmonary fibrosis (PF) as a sequela of chronic inflammation (Tobón et al., 2003). PF is observed in patients with PCM even before treatment as necroscopic findings reveal the presence of fibrosis characterized by extensive areas of collagen deposition near the hilar region and involving other structures, such as lymph nodes, bronchi, and arteries. Furthermore, collagen fibers are found on the periphery of granulomas and extend to nearby bronchi and blood vessels (Tuder et al., 1985). Fibrotic sequelae alter respiratory function and may incapacitate patients (Mendes et al., 2017). Usually, fibrotic sequelae is observed disproportionately in patients with ventilation/perfusion and alveolar-capillary blockade causing dyspnea (Campos and Cataneo, 1986). The most common abnormalities are architectural distortion and interlobular septal thickening and reticulate (Costa et al., 2013) with residual lesions occurring in up to 53% of treated patients (Tobón et al., 2003). Furthermore, emphysema, possibly due to smoking, may also be present in these patients. As a result of all these changes, an obstructive pattern is observed in lung function tests (Lemle et al., 1983).

The development of PF is generally related to a dysregulation of wound healing (Witte and Barbul, 1997). During homeostatic wound healing, fibroblasts proliferate and produce factors related to tissue repair, such as transforming growth factor beta 1 (TGF-β1), which act in paracrine and autocrine fashions to induce the differentiation of fibroblasts to myofibroblasts (Thannickal et al., 2003; Midwood et al., 2004). Myofibroblasts are cells involved in the production of extracellular matrix, fibronectin, and collagen and are characterized by the expression of alpha smooth actin (α-SMA), a protein that integrates actin filaments and proportionate the contractile phenotype of these cells (Hinz et al., 2001; Peyton et al., 2008). Although beneficial in the beginning, tissue repair can become pathogenic if it occurs rampantly, resulting in extracellular matrix remodeling and permanent scarring.

Evaluation of PF in PCM has been limited. A well-established granuloma surrounded by intense deposition of collagen type I and III in the lung parenchyma is typically observed in *P. brasiliensis*-infected mice after 4–8 weeks of fungal inoculation (Cock et al., 2000). Finato et al. (2020) also observed high concentrations of profibrotic mediators, such as IL-6, IL-1β, CCL3, IL-10, TGF-β1, VEGF, and interferon (IFN)-γ in the lungs of *P. brasiliensis*-infected mice on the eighth week of infection. High production of TGF-β1 and bFGF by *P. brasiliensis* exoantigens-stimulated monocytes from monocytes of untreated CF PCM patients has been described (Venturini et al., 2014). Araujo et al. (2011) verified PF in patients that do not received treatment. Furthermore, Tuder et al. (1985) verified the proliferation of reticular fibers in remote areas of granulomatous reactions, leading to the hypothesis that fungal components may promote collagen production. Despite this evidence, the interaction between *Paracoccidioides* spp. and pulmonary fibroblasts has not yet fully elucidated.

On the other hand, the interaction of *P. brasiliensis* and host cells has been investigated regarding the mechanisms involved in adherence and escape (Vicentini et al., 1994; Mendes-Giannini et al., 2008; Ywazaki et al., 2011). Vicentini et al. (1994) demonstrated that extracellular matrix protein laminin binds specifically to yeast forms of *P. brasiliensis* and enhances adhesion of the fungus to the surface of epithelial cells. Ywazaki et al. (2011) showed the adhesion of *P. brasiliensis* to GM1 and GM3 gangliosides of human pulmonary fibroblasts, which may be the path of bound/infection by the fungus. In another study, Mendes-Giannini et al. (2008) reported interactions between *P. brasiliensis* and epithelial Vero and A549 cells and suggested that the adhesion and invasion of these cells could represent an escape mechanism and contribute to the spread of infection.

Considering the possible influences of *Paracoccidioides* spp. on the activity of pulmonary fibroblasts and development of fibrosis in lungs, we investigated the influence of *Paracoccidioides* spp. exoantigens on the proliferation and responsiveness of human and murine pulmonary fibroblasts.

## Materials and Methods

### *Paracoccidioides* spp. Isolates

The *Paracoccidioides* spp. isolates were obtained from mycology collection of Laboratório de Pesquisa em Moléstias Infecciosas (UNESP, Botucatu, SP, Brazil). Were used in the study *P. brasiliensis* isolates Pb18 and Pb326, isolated from a patient from Botucatu, SP, Brazil, and *P. lutzii* isolates Pb01, Pb66, and Pb8334. The isolates were maintained by frequent subculture at 36°C in semi-solid GPY media containing 2% glucose, 1% peptone, 0.5% yeast extract, and 2% agar.

### Exoantigen Production

Total exoantigen was produced according to Camargo et al. (2003) with minor modifications. Briefly, yeast forms of *Paracoccidioides* spp. were subcultured in Sabouraud broth containing 2% dextrose (Sigma-Aldrich, St. Louis, MO, USA) and supplemented with 0.01% thiamine (Sigma-Aldrich) and 0.14% L-asparagine (Sigma-Aldrich) and maintained at 37°C for 3 days. The fungi were cultivated in supplemented Sabouraud broth for 3 days shaking at 50 rpm at 37°C. Next, more supplemented Sabouraud broth was added and the cultures cultivated for 7 more days at 37°C with shaking at 50 rpm. The fungi were killed by the addition of sodium merthiolate (0.2 g/L) for 24 h at 4°C and filtered using Whatman™ filter paper #1. The filtrate was dialyzed against several changes of distilled water for 24 h at 4°C. The dialysate was then filtered and concentrated by centrifugation at 4,000 rpm for 30 min at 4°C using an Amicon^®^ Ultra 15 Filter (Millipore, Billerica, MA, USA). Protein concentrations were determined using a Pierce™ BCA Protein Assay Kit (Thermo Fisher Scientific, Waltham, MA, USA). The gp43 protein was obtained by of *P. brasiliensis* B-339 according Saraiva et al. (1996) and kindly provided by Dr. Zoilo Pires de Camargo (Federal University of São Paulo, UNIFESP, Brazil).

### Human Lung Fibroblasts

The human lung fibroblast cell line MRC5 (ATCC CCL-171) was purchased from Banco de Células do Rio de Janeiro, RJ, Brazil. Fibroblasts were incubated at 37°C in a humidified 5% CO_2_/95% air atmosphere in complete Dulbecco’s Modified Eagle Medium (DMEM; Sigma-Aldrich) containing 10% fetal bovine serum (FBS; Sigma-Aldrich), 100 U/ml penicillin, and 100 µg/m; streptomycin (Sigma-Aldrich). When the cell cultures reached 80% confluence, the cells were dispersed using trypsin-EDTA (Sigma-Aldrich) for 5 min and then transferred to new culture flasks (Greiner BioOne, Frickenhausen, BW, GER).

### Isolation of Murine Pulmonary Fibroblasts

Pulmonary fibroblasts were isolated from mice using their differential adherent properties as described previously (Trentin et al., 2015; Verma et al., 2016) with modifications. Male BALB/c mice, 4 weeks old, were obtained from Instituto Lauro de Souza Lima, Bauru, SP, Brazil. All mice received a sterile balanced diet and water *ad libitum* and were kept in a ventilated shelf ALERKS-56 housing system (Alesco^®^, Monte Mor, SP, Brazil). The experimental protocol was performed in accordance with the ethical principles for animal research adopted by the National Council for the Control of Animal Experimentation (CONCEA). Briefly, *naïve* young mice (4-week-old) were euthanized by intraperitoneal administration of ketamine and xylazine. After thoracotomy under aseptic conditions, the lungs were perfused with sterile phosphate-buffered saline. The perfused lungs were then removed and cut into small pieces and underwent two rounds of enzymatic digestion using collagenase type II (Gibco, Life Technologies, Paisley, UK) and trypsin (0.25%; Gibco, Life Technologies). Cell viability was determined using 0.1% trypan blue staining. The cells were then aliquoted into 25 cm^2^ cell culture flasks (Corning Costar, New York, NY, USA) at a proportion of 1 lung/flask in 1 ml. Then, 4.0 ml of DMEM supplemented with 20% fetal calve serum was added and the cells incubated at 37°C with 5% CO_2_ in a humidified chamber. After 24 h, the medium was changed to remove non-adherent cells. Once the cell culture reached 70% confluence, they were dispersed using trypsin-EDTA (Sigma-Aldrich) for 5 min and then resuspended in supplemented DMEM. Fibroblast isolation was confirmed using by immunofluorescence staining based on CD90 expression (**Supplementary Figure 1**). Fibroblasts were used after two to three passages.

### Fibroblast Cell Cultures

Human and murine fibroblasts were incubated with exoantigens of *P. brasiliensis* and *P. lutzii* at concentrations of 2.5, 5, 10, 100, and 250 µg/ml and gp43 at concentrations of 5 and 10 µg/ml. The protein gp43 is the immunodominant antigen of *P. brasiliensis*. (Puccia et al., 1986). After 24 h, the cells were analyzed using proliferation assays and cell-free supernatants were evaluated for cytokines.

### Proliferation Assays/Viability Assay

Cell viability and proliferation were measured using MTT assays according to Mosmann (1983). Briefly, fibroblasts were seeded into 96-well culture plates in octuplicate at 2 × 10^4^ murine pulmonary fibroblast/well and 1 × 10^4^ human pulmonary fibroblasts/well. After 24 h of incubation, the cells were stimulated with *Paracoccidioides* spp. exoantigens. At 24-h post-stimulation, the supernatants were collected and the cells then incubated in complete DMEM containing MTT (5 mg/ml). The plate was incubated for 2 h at 37°C in 5% CO_2_ and then centrifuged for 5 min at 1,500 rpm. The supernatants were removed and the cells in each well resuspended in 100 ml of dimethyl sulfoxide (DMSO). After 5 min, the plate was read at 540 nm using a spectrophotometer reader. The percentage of proliferation was calculated according to the ratio between the treated and control cultures multiplied by 100.

### Functional Analyses

Levels of human basic fibroblast growth factor (bFGF), TGF-β1, interleukin (IL)-1β, tumor necrosis factor alpha (TNF-α), and pro-collagen I and mouse levels of TGF-β1, IL-1β, IL-6, and vascular endothelial growth factor (VEGF) were measured in the cell-free supernatants using a Duo-Set Kit (R&D Systems, Minneapolis, MI, USA), according to the manufacturer’s instructions. Results were expressed as pg/ml and determined using standard curves established for each assay.

### Ethical Aspects

The study was approved by the Research Ethics Committees of Botucatu Medical School, UNESP (CAEE #62177516.3.0000.5411) and the experimental approach was approved by the Ethical Committee of School of Sciences (Proc. #760/2016; UNESP, Bauru, São Paulo, Brazil).

### Statistical Analyses

The two matched groups were compared using the paired t-test. Multiple group comparisons were performed using one-way analysis of variance (ANOVA) with Dunnet’s *post hoc* tests. All statistical analyses were performed using GraphPad Prism 5.0 software (GraphPad Software. Inc., San Diego, California, USA) at a significance level of 5% (p < 0.05) (Zar, 2010).

## Results

### *Paracoccidioides* spp. Exoantigens Induced Enhanced Proliferation in Murine Pulmonary Fibroblasts and Discrete Proliferation in Human Pulmonary Fibroblasts

**Figure 1:**
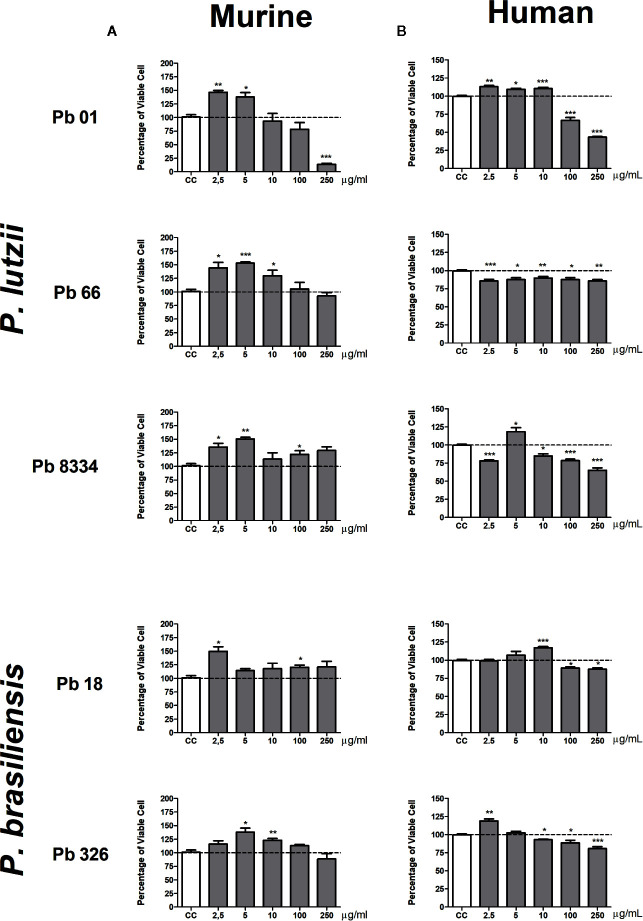
Percentage of viable pulmonary fibroblasts stimulated with *Paracoccidioides* spp. exoantigens. Fibroblasts were cultured in the presence or absence of *Paracoccidioides* spp. exoantigens and evaluated 24 h post-treatment using MTT assays. **(A)** Murine pulmonary fibroblasts. **(B)** Human pulmonary fibroblasts. Fibroblast proliferation was measured according to the ratio of test culture cells (challenged with exoantigens) to untreated culture control (CC) cells. Values above 100% of viability represent cell proliferation. Results are expressed as mean ± SEM; paired t-test, *p < 0.05, **p < 0.01, ***p < 0.001; n = 4.

The behavior of murine pulmonary fibroblasts was similar when stimulated with the exoantigens prepared from different *Paracoccidioides* species and isolates. Exoantigens from all *P. lutzii* isolates (Pb01, Pb66, and Pb8334) induced cell proliferation at the lower concentrations of 2.5 and 5 µg/ml represented by increase percentage of viable cell compared to non-stimulated culture (**Figure 1A**). Isolate Pb8334 exoantigen also promoted cell proliferation at 100 µg/ml and the exoantigen of Pb66 promoted cell proliferation at 10 µg/ml. The exoantigen of isolate Pb01 was cytotoxic at the higher concentration of 250 µg/ml. *P. brasiliensis* exoantigens also promoted cell proliferation at different concentrations depending on the particular isolate. For instance, Pb18 exoantigen promoted cell proliferation at 2.5 and 100 µg/ml, whereas Pb326 exoantigen promoted proliferation at 5 and 10 µg/ml. Neither of the *P. brasiliensis* exoantigens was cytotoxic to murine pulmonary fibroblasts at the concentrations tested (**Figure 1A**).

Human pulmonary fibroblasts were more sensitive to *Paracoccidioides* spp. exoantigens compared to that of murine fibroblasts, exhibiting discrete proliferation and cytotoxicity at the higher concentrations (100 and 250 µg/ml) of both species tested (**Figure 1B**). Pb01 exoantigen induced cell proliferation at the lowest concentrations tested of 2.5, 5, and 10 µg/ml. In contrast, Pb18 exoantigen induced proliferation at only 5 µg/ml. For the two *P. brasiliensis* isolates, Pb18 exoantigen induced cell proliferation at 10 µg/ml, while Pb326 exoantigen induced cell proliferation at a concentration of 2.5 µg/ml but was cytotoxic at 10 µg/ml (**Figure 1B**).

### *Paracoccidioides* spp. Exoantigens Decreased IL-6 and VEGF Production by Murine Pulmonary Fibroblasts

**Figure 2:**
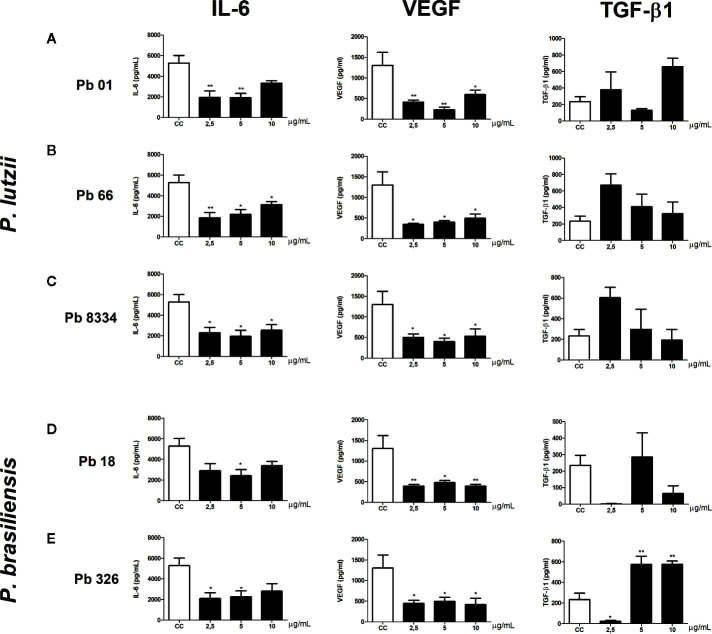
Murine pulmonary fibroblast activity stimulated by *Paracoccidioides* spp. exoantigens. Fibroblasts were cultured in the presence or absence of *Paracoccidioides* spp. exoantigens and levels of IL-6, VEGF, and TGF-β1 in the cell-free supernatants were determined 24 h post-treatment. **(A)** Pb01 exoantigen. **(B)** Pb66 exoantigen. **(C)** Pb8334 exoantigen. **(D)** Pb18 exoantigen. **(E)** Pb326 exoantigen. Results are expressed as means ± SEM; ANOVA with Dunnett’s *post hoc* test; *p < 0.05, **p < 0.01; n = 4.

To evaluate the effects of exoantigens of the activity of murine pulmonary fibroblast, we measured the levels of cytokines and growth factors involved in inflammation and wound healing. All exoantigens of *P. brasiliensis* and *P. lutzii* caused decreased levels of VEGF compared to that of the control group at all exoantigen concentrations tested (**Figure 2**). Also, decreased levels of IL-6 were observed in murine pulmonary fibroblasts stimulated with 2.5 and 10 µg/ml of Pb01 and Pb66 exoantigens (**Figures 2A, B**) and 2.5, 5, and 10 µg/ml of Pb8334 exoantigen (**Figure 2C**) compared to that of the control group. Exoantigen from the *P. brasiliensis* isolate Pb18 at 5 µg/ml induced decreased levels of IL-6 (**Figure 2D**). Meanwhile, compared with that of the control group, exoantigen from the *P. brasiliensis* isolate Pb326 was also shown to decrease levels of IL-6 at 2.5 and 5 µg/ml as well as decrease levels of TGF-β1 at 2.5 µg/ml, but increased levels of TGF-β1 at 5 and 10 µg/ml (**Figure 2E**). Levels of IL-1β were not detected in the supernatants of murine fibroblast cultures.

### Intense Production of Pro-collagen I and TGF-β1 by Human Pulmonary Fibroblast Was Induced by *Paracoccidioides* spp. Exoantigens

**Figure 3:**
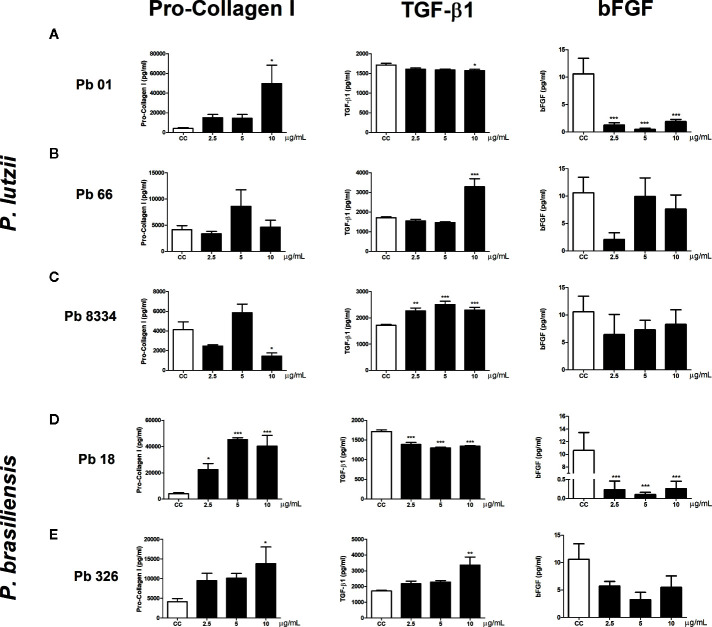
Human pulmonary fibroblast activity stimulated by *Paracoccidioides* spp. exoantigens. Fibroblasts were cultured in the presence or absence of *Paracoccidioides* spp. exoantigens and levels of pro-collagen I, TGF-β1, and bFGF in the cell-free supernatants were determined 24 h post-treatment. **(A)** Pb01 exoantigen. **(B)** Pb66 exoantigen. **(C)** Pb8334 exoantigen. **(D)** Pb18 exoantigen. **(E)** Pb326 exoantigen. Results are expressed as means ± SEM; ANOVA with Dunnett’s *post hoc* test; *p < 0.05, **p < 0.01, ***p < 0.001; n = 4.

Functional analyses of human pulmonary fibroblasts compared to that of non-stimulated cells (control group) revealed intense pro-collagen I production in cells stimulated with Pb01 exoantigen at 10 µg/ml (**Figure 3A**), Pb18 exoantigen at 2.5, 5, and 10 µg/ml (**Figure 3D**), and Pb326 exoantigen at 10 µg/ml (**Figure 3E**). Decreased pro-collagen I production was also seen in cells stimulated with 10 µg/ml of Pb8334 exoantigen compared to that in non-stimulated cells (**Figure 3C**). No difference in pro-collagen I production was observed in cells stimulated with Pb66 exoantigen. In addition, increased TGF-β1 production was detected in cells stimulated with 10 µg/ml Pb66 exoantigen (**Figure 3B**), 2.5, 5, and 10 µg/ml Pb8334 exoantigen (**Figure 3C**), and 10 µg/ml Pb326 exoantigen (**Figure 3E**) compared to that in the control group. In contrast, decreased TGF-β1 levels was seen in cells stimulated with 10 µg/ml Pb01 exoantigen (**Figure 3A**) and 2.5, 5, and 10 µg/ml Pb18 exoantigen (**Figure 3D**). Decreased bFGF levels were observed in fibroblasts stimulated with Pb01 exoantigen (**Figure 3A**) and 2.5, 5, and 10 µg/ml Pb18 exoantigen (**Figure 3D**). IL-1β was not detected in the supernatants of human pulmonary fibroblasts.

### Gp43 Was Cytotoxic and Increased TGF-β1 Levels Only in Human Pulmonary Fibroblasts

**Figure 4:**
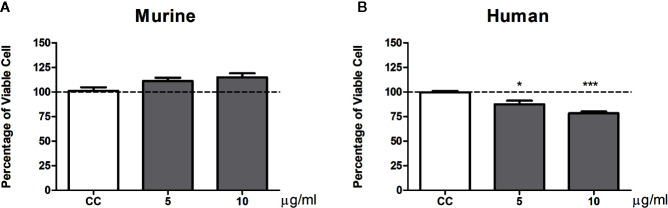
Percentage of viable pulmonary fibroblasts stimulated by gp43. Fibroblasts were cultured in the presence or absence of gp43 and proliferation evaluated 24 h post-treatment using MTT assays. **(A)** Murine pulmonary fibroblasts. **(B)** Human pulmonary fibroblasts. Fibroblast proliferation was measured according to the ratio of test culture cells (challenged with exoantigens) to untreated culture control (CC) cells. Results are expressed as mean ± SEM; paired t-test; *p < 0.05, ***p < 0.001; n = 4.

**Figure 5:**
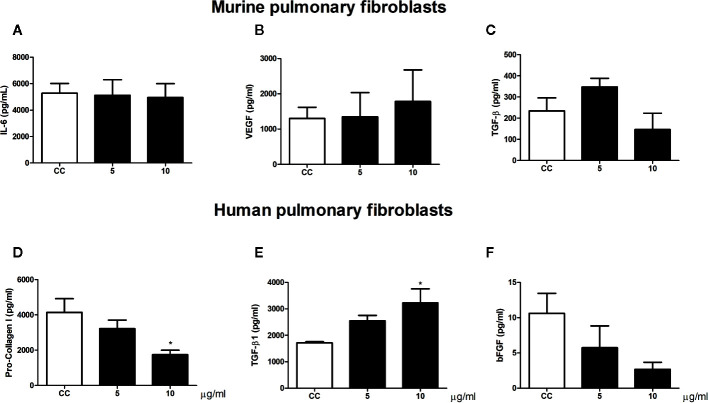
Pulmonary fibroblast activity stimulated by gp43. Human and murine fibroblasts were cultured in the presence or absence of gp43 and IL-6, VEGF, TGF-β1, pro-collagen I, and bFGF levels in the cell-free supernatants were determined 24 h post-treatment. **(A)** IL-6. **(B)** VEGF. **(C)** TGF-β1. **(D)** Pro-collagen I. **(E)** TGF-β1. **(F)** bFGF. Results are expressed as means ± SEM; ANOVA with Dunnett’s *post hoc* test; *p < 0.05; n = 4.

We also analyze the influence of gp43 on pulmonary fibroblast function. No differences in viability (**Figure 4**) or cytokine production (**Figure 5**) were observed in murine pulmonary fibroblasts at any concentration of gp43 tested. No IL-1β was not detected in the supernatants of mouse pulmonary fibroblasts. In human pulmonary fibroblasts, gp43 was cytotoxic at 5 and 10 µg/ml compared to that in non-stimulated cells (**Figure 4**). Decreased pro-collagen I levels and increased TGF-β1 production were also observed in human pulmonary fibroblasts treated with 10 µg/ml gp43 (**Figures 5A–B**). No significant difference was observed in bFGF production. Consistent with the supernatants of mouse pulmonary fibroblasts, no IL-1β was not detected in supernatants of human pulmonary fibroblasts.

## Discussion

In the current study, we showed for the first-time interactions of *Paracoccidioides* spp. exoantigens with human and murine pulmonary fibroblasts. Exoantigens of both *Paracoccidioides* species, *P. lutzii* and *P. brasiliensis*, induced proliferation of human and murine fibroblasts. Interestingly, in murine pulmonary fibroblasts *P. lutzii* isolates Pb01 and Pb66 and *P. brasiliensis* isolate Pb326 increased cell proliferation at the lowest concentrations of exoantigen tested. This was in conjunction with reduced viability of cells stimulated with the higher concentrations of the exoantigens, suggesting a dose-dependent effect. In human pulmonary fibroblasts, we also observed increased proliferation induced by the lowest concentrations of exoantigens; however, Pb66 exoantigen reduced cell viability at every concentration tested. Pb18 and Pb8334 exoantigens seemed to not cause the same effect as the exoantigens from the other isolates. These exoantigens induced cell proliferation at different concentrations, but without it being a dose-dependent effect. Diversity among isolated *Paracoccidioides* species has been previously explored. For instance, Machado et al. (2013) reported differences in exoantigen composition of Pb01, Pb8334, Pb18, Epm83, and Pb265 isolates. They also demonstrated that *P. lutzii* expresses lower amounts of gp43 compared to that of *P. brasiliensis*. Furthermore, de Oliveira et al. (2018) analyzed the secretome of isolates Pb01 and Epm83 and showed that isolates of the *Paracoccidioides* complex are able to secrete different proteins, mainly those related to adhesion and virulence.

The mainly pathological pulmonary characteristic in CF-PCM is the granulomatous inflammatory process (Queiroz-Telles and Escuissato, 2011) with lesions typically surrounded by fibroblasts and collagen fibers after 2–3 weeks of fungal infection (Kerr et al., 1988; Cock et al., 2000). Fibroblasts are directly involved with collagen production and the establishment of fibrosis during chronic inflammation (Wick et al., 2013). Therefore, our results provide evidence that *Paracoccidioides* spp. may influence the modulation of pulmonary fibroblasts by inducing cell proliferation and acting directly on the development of non-regulated wound healing, which could support the establishment of fibrosis. Other investigators suggest other main roles for fibroblasts and myofibroblasts during infections. For instance, the pathophysiological basis of inflammatory myofibroblastic tumors (IMTs) is related to an uncontrolled response to tissue damage or chronic inflammation. Furthermore, the development of IMTs have previously been related to chronic inflammation caused by histoplasmosis (Cassivi and Wylam, 2006). Rosa et al. (2019), using an experimental model, showed that *Cryptococcus gatti* infection increases the expression in the lungs of proteins related to energy metabolism, leading to activation of the glycolytic pathway. In the same study, these authors confirmed the activation of glycolysis in human pulmonary fibroblasts, culminating in the Warburg effect (WE). Interestingly, WE is known for its involvement with cell proliferation, mainly tumor cells. A similar change has been observed in a murine model of pulmonary infection by *Mycobacterium tuberculosis* in which the authors described as an infection-induced WE (Shi et al., 2015). Furthermore, an *in vitro* study showed that *M. tuberculosis* stimulates murine lung fibroblasts to proliferate and differentiate into myofibroblasts (Verma et al., 2016).

Fibroblasts play a key role in the tissue repair process, but may also impact immune responses (Buechler and Turley, 2018). In rheumatoid arthritis, IL-6 produced by synovial fibroblasts contributes to the autoimmunity associated with this disease (Nguyen et al., 2017; Buechler and Turley, 2018). Meanwhile, lung fibroblasts can recruit dendritic cells into airway lymph nodes through an integrin-mediated inflammatory signaling (Kitamura et al., 2011; Buechler and Turley, 2018). Liver fibroblasts infected by *Leishmania donovani* induce the generation of T-regulatory (Treg) lymphocytes. The depletion of liver fibroblasts *in vivo* reduces the number of Treg lymphocytes and decreases the parasitic burden (Khadem et al., 2016; Buechler and Turley, 2018). Patients with CF-PCM commonly present with lung fibrosis and persistent nonspecific inflammatory responses (Mendes et al., 2017). Similar to PCM, other infectious agents can cause chronic inflammation and the establishment of fibrosis. In necropsies of acquired immunodeficiency syndrome (AIDS) patients with pneumocystosis, it is possible to detect chronic inflammation and interstitial fibrosis in the lungs (Foley et al., 1993). In chronic pulmonary histoplasmosis, it is common for pulmonary inflammation to lead to fibrosis and volume loss with compensatory enlargement of cavities and pleural thickening. In rare cases, mediastinal fibrosis may occur, which is an abnormal and exuberant post-infection fibrotic response for which the mechanism has not been elicited (Wheat et al., 2016).

To investigate the link between immune responses and fibrosis in PCM, we evaluated fibroblasts for the production of targeted cytokine related to inflammation and tissue repair. In murine cells, we determined that all *Paracoccidioides* exoantigens promoted decreased levels of IL-6 and VEGF, but interestingly Pb326 exoantigen induced increased levels of TGF-β1. In human pulmonary fibroblasts, we also observed increased levels of TGF-β1 produced by cells stimulated with Pb66, Pb8334, and Pb326 exoantigens and increased levels of pro-collagen I produced by cells stimulated with Pb01, Pb18, and Pb326 exoantigens. We also observed increased proliferation of human fibroblasts, but bFGF levels were lower than that of the untreated control cells. Venturini et al. (2014) showed that *P. brasiliensis* antigens increase the production of IL-1β, TNF-α, TGF-β1, and bFGF by peripheral blood monocytes of CF-PCM patients, suggesting fungal metabolites may play an important role in the activation of these cells. Therefore, *Paracoccidioides* spp. exoantigens may also participate in the establishment of fibrosis in PCM. This could occur by activating monocytes to produce high levels of bFGF and TGF-β1 and activating fibroblasts to produce TGF-β1 and pro-collagen I. TGF-β1 is a potent pro-mitotic factor that contributes to increased collagen production and others extracellular matrix compounds (Midwood et al., 2004) and is also involved in the transformation of fibroblasts into myofibroblasts (Thannickal et al., 2003). Pulmonary fibrosis is characterized by the loss of lung epithelial cells and the proliferation of fibroblasts (Mendes-Giannini et al., 2008). Also, *P. brasiliensis* may modulate apoptosis of epithelial cells A549 by the expression of apoptotic molecules such as Bcl-2, Bak, and caspase-3, confirming the inducing of apoptosis by the fungus which can then survive and spread to other parts of the body (Del Vecchio et al., 2009). This suggests that exoantigens may have a role in the mechanisms that regulate fibrosis in PCM. An interesting fact regarding *Paracoccidioides* spp. is that they are capable of surviving in hypoxic environments (Lima et al., 2015), such as granulomatous centers. Hypoxia directly influences wound repair by activation of HIF-1α, transformation of fibroblasts into myofibroblasts, increased expression of α-SMA, collagen I, and collagen III, and activation of SMAD3 (Zhao et al., 2017). Mendes et al. (2017) discuss the pathogenesis and the role of host’s immune in PCM, and one of the cases is when after infection, the inflammatory reaction recedes and scars are formed, which may be sterile or contain viable, albeit latent fungi. So, the fungi may remain latent for many years, however, any imbalance may result in reactivation of latent foci, a phenomenon known as endogenous reinfection, which triggers disease. Restrepo (2000) also mentions and discuss about cases where the infection results in residual foci containing viable fungi that may outcome in endogenous reactivation, as shown by cases diagnosed outside of the geographic limits of the mycosis. Taken together, this data provides evidence for *Paracoccidioides* spp. being able to potentiate tissue repair functions of fibroblasts, resulting in cell proliferation and increased collagen production, which can lead to changes in the lung structure and low oxygen circulation. This new environment would be conducive to fungal survival and escape from the immune system with the fungi being hidden for prolonged periods in granulomatous centers.

Gp43 is a high mannose glycoprotein, the main antigen secreted by *P. brasiliensis*, and is usually used as a diagnostic antigen (Flavia Popi et al., 2002). It has been shown in an experimental model that gp43 is able to inhibit the fungicidal ability of macrophages, suggesting that the avoidance mechanisms of *Paracoccidioides* spp. may favor the primary infection status of susceptible hosts (Flavia Popi et al., 2002). Therefore, we investigated the possible influences of gp43 on pulmonary fibroblasts. Our results failed to show an important role for gp43 in the induction of murine pulmonary fibroblast proliferation. However, we did observe that gp43 promoted decreased cell viability and increase TGF-β1 production in human pulmonary fibroblasts. Curiously, gp43 strongly stimulates granuloma formation in a murine model using peritoneal macrophages and B-1 cells isolated from A/J mice (Vigna et al., 2006). It is important to highlight that *P. lutzii* has an ortholog glycoprotein called Plp43 that presents a peptide sequence only of 81% identical with *P. brasiliensis* (Leitão et al., 2014). Thus, *P. lutzii* exoantigens represents a control of enriched gp43 exoantigen.

Our results require further investigation. Despite the evidence that exoantigens of *Paracoccidioides* spp. modulate the function of human and mouse pulmonary fibroblasts, we did not evaluated different antigenic components from both fungal strain. Other limitations were we did not prove if chosen concentration range has any physiological meaning, the maturation of fibroblasts into myofibroblasts, or the presence of other factors that may influence fibrogenesis, such as the downregulation of metalloproteinases. While our study may not present a comprehensive model defining all the mechanisms of how *Paracoccidioides* spp. exoantigens modulate the pulmonary fibroblasts, we believe that it is an important source to start understanding how this fungus influences tissue repair function of these cells.

In summary, our results demonstrate, for the first time, that *Paracoccidioides* spp. exoantigens may promote pulmonary fibroblast proliferation and gp43, the immunodominant antigen of *P. brasiliensis*, is not related to the stimulation of fibroblast proliferation, but may have a role in the maturation of fibroblasts into myofibroblasts. Further studies are needed to better understand the mechanistic process in greater detail.

## Data Availability Statement

The original contributions presented in the study are included in the article/**Supplementary Materials**. Further inquiries can be directed to the corresponding author.

## Ethics Statement

The animal study was reviewed and approved by National Council for the Control of Animal Experimentation.

## Author Contributions

Conception and design of the experiments: DA, TD, RC, RM, APS, and JV. Performance of the experiments: DA, KR, ARS, AF, and APS. Analysis of the data: DA and JV. Preparation of the manuscript: DA and JV. All authors contributed to the article and approved the submitted version.

## Funding

This study was financially supported in part by the Fundação da Universidade Federal do Mato Grosso do Sul, UFMS/MEC, BRASIL. This study was also supported by the Coordenação de Aperfeiçoamento de Pessoal de Nível Superior, Brasil (CAPES; Finance Code 001) and Conselho Nacional de Desenvolvimento Científico e Tecnológico (CNPq; Grant #470221/2014-3).

## Conflict of Interest

The authors declare that the research was conducted in the absence of any commercial or financial relationships that could be construed as potential conflicts of interest.
