# Supplementary material for: Exoantigens of Paracoccidioides spp. Promote Proliferation and Modulation of Human and Mouse Pulmonary Fibroblasts
Source: Front Cell Infect Microbiol. 2020 Oct 30;10:590025. doi: 10.3389/fcimb.2020.590025 (PMC7662685; doi:10.3389/fcimb.2020.590025)
Supplement: Supplementary file 1 [file DataSheet_1.pdf]

Supplementary Material

Supplementary Figure

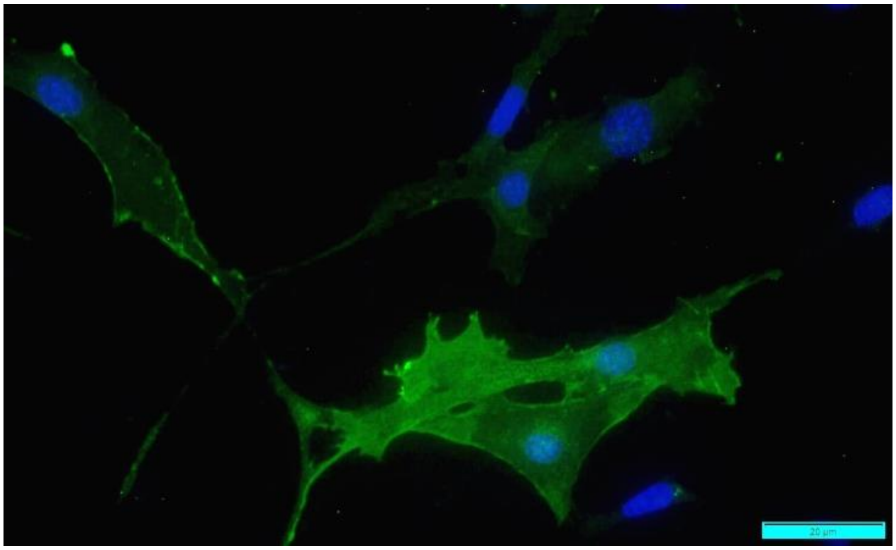

Figure S1. CD90 immunofluorescence staining. Pulmonary fibroblasts were isolated from BALB/c mice. After 8 days of isolation, cells were seeded in round coverlips for 24h and submitted to immunofluorescence (green:CD90; blue: cell nucleus labeled by DAPI). The stained cells were observed by an Olympus fluorescence microscope. Scale bar: 20 μm.

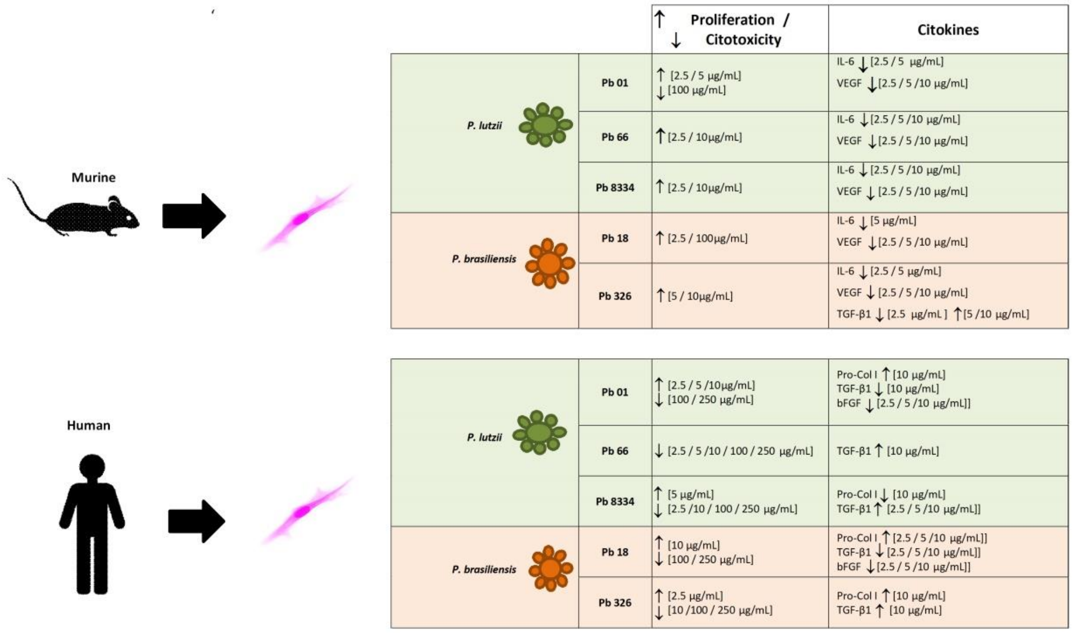

Figure S2. Overview of *Paracoccidioides* ssp exoantigens influences to murine and human pulmonary fibroblasts. Pulmonary fibroblasts were cultivated with 2.5, 5, 10, 100, and 250 µg/mL of exoantigens produced from *P. brasiliensis* (Pb18 and Pb326) and *P. lutzii* (Pb01, Pb8334 and Pb66) isolates. After 24 hours, cell proliferation and production of cytokines and grow factors by pulmonary fibroblasts were evaluated.
